# Visual habituation in deaf and hearing infants

**DOI:** 10.1371/journal.pone.0209265

**Published:** 2019-02-06

**Authors:** Claire Monroy, Carissa Shafto, Irina Castellanos, Tonya Bergeson, Derek Houston

**Affiliations:** 1 Department of Otolaryngology—Head and Neck Surgery, Ohio State University Wexner Medical Center, Columbus, Ohio, United States of America; 2 Nationwide Children’s Hospital, Columbus, Ohio, United States of America; 3 Insight Data Science, New York City, New York, United States of America; 4 Department of Communication Sciences and Disorders, Butler University, Indianapolis, Indiana, United States of America; Swinburne University of Technology, AUSTRALIA

## Abstract

Early cognitive development relies on the sensory experiences that infants acquire as they explore their environment. Atypical experience in one sensory modality from birth may result in fundamental differences in general cognitive abilities. The primary aim of the current study was to compare visual habituation in infants with profound hearing loss, prior to receiving cochlear implants (CIs), and age-matched peers with typical hearing. Two complementary measures of cognitive function and attention maintenance were assessed: the length of time to habituate to a visual stimulus, and look-away rate during habituation. Findings revealed that deaf infants were slower to habituate to a visual stimulus and demonstrated a lower look-away rate than hearing infants. For deaf infants, habituation measures correlated with language outcomes on standardized assessments before cochlear implantation. These findings are consistent with prior evidence suggesting that habituation and look-away rates reflect efficiency of information processing and may suggest that deaf infants take longer to process visual stimuli relative to the hearing infants. Taken together, these findings are consistent with the hypothesis that hearing loss early in infancy influences aspects of general cognitive functioning.

## Introduction

Infants learn about the world through their multimodal interactions with objects and other people in their environment [1–3]. The atypical functioning of one sensory system may result in widespread effects across multiple sensory modalities and cognitive domains [4,5]. The auditory system, in particular, is thought to play a pivotal role in shaping the cognitive system [6]. As a result, researchers have recently begun to investigate the impact of prelingual hearing loss on cognition from a developmental perspective [7,8].

Several studies have shown that deaf children exhibit poorer performance in multiple nonverbal cognitive skills compared to their hearing peers. These include visual controlled attention (e.g., [9]), sequence processing [10,11], and working memory [12]. Other studies have also reported differences in motor abilities that require cognitive skills, such as in spatial coordination [13] and visual-motor integration skills [14]. Together, these findings suggest that general cognitive abilities differ between deaf and hearing children. However, the underlying sources of these differences are still unknown, as is when the differences emerge during infancy.

Differences between deaf and hearing individuals may initially arise from cortical cross-modal re-organization. In the absence of sound input, the auditory cortex responds to visual and somatosensory input [7,15,16]. Visual cross-modal re-organization has also been demonstrated in children as young as five years of age [17]. Cross-modal plasticity is thought to arise via compensatory mechanisms of the remaining senses following a sensory loss. However, evidence for poorer performance in deaf individuals has contributed to sensory deprivation hypotheses, which stem from the assumption that our sensory systems are intrinsically multimodal [1]. Atypical functioning in one modality will therefore have cascading consequences throughout other sensory modalities. Given the evidence that multimodal sensory processing underlies cognition (e.g., embodied cognition theories), atypical sensory functioning should also affect cognitive abilities.

One example, proposed by Smith and colleagues [18], is the hypothesis that hearing loss causes poorer multimodal sensory integration, which, in turn, causes deficits in visual selective attention and cognitive control. These authors suggest that multisensory integration is essential for the development of attentional skills in each individual sensory modality. In support of this hypothesis, they report data showing that cognitive control and visual selective attention in deaf children improves following cochlear implantation [18]. Another example of a sensory deprivation hypothesis is the auditory scaffolding hypothesis, which emphasizes the importance of auditory input for typical development of cognitive functions [19]. According to this hypothesis, sound provides experience with naturally sequential input that is vital for developing general sequence learning abilities. Sequence learning, in turn, influences a variety of other domain-general cognitive abilities and can have widespread consequences on multiple aspects of development.

An alternate explanation is that deaf children’s performance on cognitive and attentional tasks differs from that of hearing children because of their limited language experiences, rather than general cognitive abilities [20–22]. Early language and communicative experiences are critical for the typical development of social and cognitive skills [23]. However, the majority of deaf infants experience mismatched communication exchanges with their hearing parents—that is, the reciprocal communication pattern in typical parent-infant interactions is disrupted when the infant’s hearing status does not match that of their parent—and therefore experience difficulties in language development [24,25]. As a result, poor performance on attentional tasks could arise from early language delays [21]. This hypothesis is supported by evidence from Deaf children of Deaf parents (DOD)—children who are exposed to native American Sign Language (ASL) from birth and achieve typical social and language milestones [26].

In sum, a growing body of work provides evidence for differences in nonverbal cognitive abilities between deaf and hearing children, but the underlying sources remain unclear. Most of the research described above targeted preschool or school-age children, but no study has yet investigated the effects of hearing loss on cognitive abilities in young infants. As a result, little is known about when these differences between deaf and hearing children emerge.

To address this gap, the current study aimed to compare visual habituation in infants with prelingual hearing loss and infants with typical hearing. Habituation is one of the earliest cognitive processes to emerge in development (for a review, see [27]). Visual habituation reflects a basic form of learning: once a stimulus is fully encoded, the infant habituates to it and demonstrates decreased attention to the stimulus. Researchers have found that the duration of time to habituate in infancy accounts for up to 30 percent of the variance in cognitive ability at older ages [28–31]. This indicates that visual habituation relates to the development of more complex cognitive abilities. In addition, evidence from prior studies suggests that ‘look-away rate’—brief gaze shifts away from the target stimulus during habituation—reflects processing efficiency and attentional control [32]. Higher look-away rates correspond to shorter look durations, and prior research has found that infants who demonstrate shorter looks during habituation are faster and more efficient at encoding information [33].

If hearing loss is associated with differences in the early development of general cognitive abilities, we predicted that deaf infants may require more trials to reach habituation criteria, have slower habituation rates (the amount of decrease in looking time from one trial to the next) and have lower look-away rates than hearing infants. To test these predictions, we compared the number of trials to reach the habituation criteria, growth slopes during habituation, and look-away rate between deaf and hearing infants using a visual habituation-oddity paradigm [34]. For the deaf infants, we also examined potential relations between visual habituation and performance on standard language assessments before and after cochlear implantation. In doing so, we aimed to provide new evidence for the relations between hearing loss and processing of non-auditory stimuli at earlier ages than previously investigated. By testing young infants prior to the onset of advanced language development, we also aimed to shed light on the discussion of whether performance differences in deaf children may be due to domain-general processing or language experiences.

## Method

### Participants

#### Deaf infants

Deaf infants were diagnosed with bilateral severe-to-profound sensorineural hearing loss. All were scheduled to receive a CI or had a CI activated within 24 hours of participation in this study (Table 1). All parents of deaf infants had self-reported typical hearing. Our final sample consisted of 23 infants (11 female) who ranged in age from 7.8 to 21.8 months at the time of testing (*M* = 13.4, *SD* = 4.4). This range reflects the typical age range in which congenital deafness was identified in our population at the time of testing. For 19 of the infants, the etiology of hearing loss was unknown. The other four infants had a diagnosis of Mondini syndrome. Twenty-one of the infants used bilateral hearing aids. Sixteen infants came from families that used oral communication only and seven infants came from families that also used a signed language. Two infants had already received a CI: one was activated on the day of testing and one was activated one day prior to testing. Twenty-three additional deaf infants were tested (16 female) but excluded from analyses because of fussiness (n = 8), they fell asleep (n = 4), had known cognitive delays (n = 7), or did not meet the habituation criterion (n = 4; see section 2.3). This study was approved by the IRB committee of Indiana University.

**Table 1:** Characteristics of the deaf infants.

	M	SD	Range
Age at Amplification (n = 15*)	4.69	2.50	1.25–10.99
Age at Test	13.38	4.36	7.86–21.84
Age at CI Activation	15.72	4.25	9.87–24.21
Years of Maternal Education	13.96	2.06	12–20
Aided PTA thresholds	79.78	13.91	47–90
Unaided PTA thresholds	107.20	12.92	78.33–120
GDQ standard score	98.71	10.65	75–121
Mental Index composite score	95.77	6.75	82–110

Note: DAYC = General Developmental Quotient from the Developmental Assessment of Young Children.

*The age at amplification was only available for 15 of the 23 infants in our sample because the remaining 7 infants did not use any form of amplification prior to cochlear implantation.

Prior to participation in the study, all deaf infants underwent the typical evaluation for a CI at Riley Children’s Hospital in Indianapolis, Indiana. This included two standardized cognitive measures: The General Developmental Quotient from the Developmental Assessment of Young Children [35] and the Mental Index score from the Bayley Scales of Infant Development [36]. Infants with scores below 70 (two standard deviations below the normative mean of 100) on either test were excluded from analyses (*n* = 7) because it may indicate significant developmental, neurological, or cognitive delays in addition to hearing loss.

#### Hearing infants

Each infant in the deaf group was matched to a typically-developing hearing infant based on chronological age (+/- 1 month). The hearing group consisted of 23 infants (14 female) who ranged in age from 7.6 to 22.7 months (*M* = 13.4, *SD* = 4.5) on the day of testing. Seventeen additional infants were tested (eight female) whose data were not included in the analyses because they did not complete testing (*n* = 15) or failed to habituate (*n* = 2). All hearing infants passed a newborn hearing screening, had no history of recurrent acute or chronic otitis media, and had no known developmental delays. Parents of all infants (deaf and hearing) provided informed written consent prior to inclusion in the study.

### Stimuli

**Fig 1:**
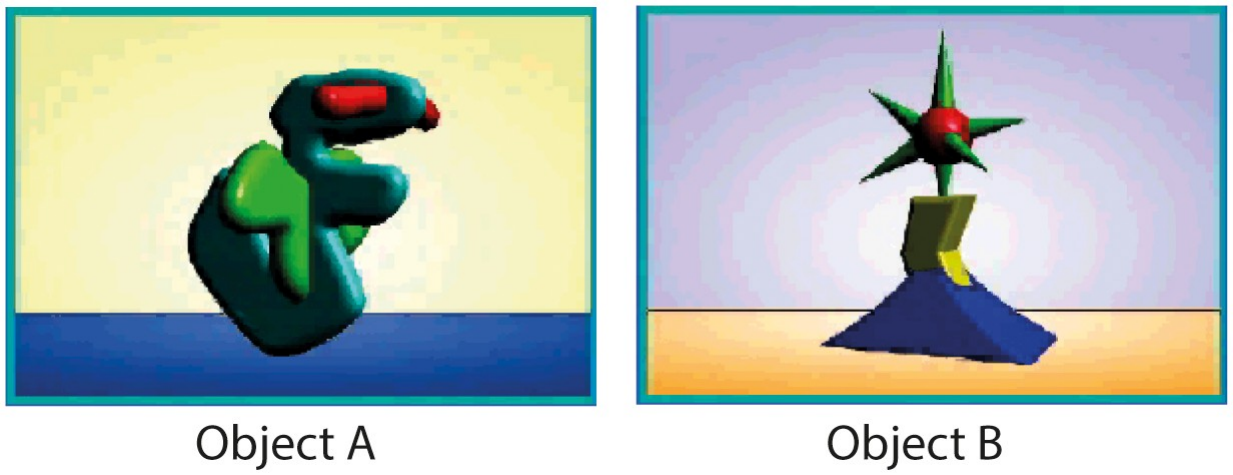
The stimuli used in the habituation task. Object A [left] served as the habituation stimulus for half of the infants and Object B [right] served as the habituation stimulus for the other half. The remaining object served as the novel stimulus.

The habituation stimuli were two images of unique and colorful unfamiliar objects (Fig 1). Each infant saw one object during habituation and the other appeared as the novel stimulus during the test phase. The stimuli were counterbalanced across infants.

We also used an attention-grabber and a pre/post-test stimulus. The attention-grabber was a silent video of an infant laughing, which was presented in the center of the screen before each trial to orient infants to the screen. The pre/post-test stimulus was a graphic animation of a blue and white geometric shape that loomed back and forth. These were unrelated to the experimental stimuli.

### Procedure

The current experiment implemented a visual habituation-oddity paradigm [34]. Unlike standard habituation procedures, in which infants view equal numbers of novel and familiar stimuli following the habituation phase, the oddity paradigm is characterized by presenting four novel and 10 familiar trials.

Infants were tested in a custom-designed double-walled sound booth. Infants sat on their caregiver’s lap approximately five feet from a 55-inch wide-aspect screen. Stimuli were displayed in the center of the screen at approximate eye level to the infants. The experimenter observed the infants from a separate room via a hidden, closed-circuit digital camera, and controlled the experiment using the Habit software package [37] on a Macintosh computer. Parents were instructed to keep their head down and refrain from interacting with their child during testing. The experimenter was blind to the stimulus condition and pushed a button on a computer keyboard whenever the infants’ eyes were oriented toward the stimulus on the screen.

A pre-test and post-test were conducted immediately before and after the experiment. Infants were presented with the geometric animation (see *Stimuli*) to measure their general visual attention over the course of the experiment. This is a method used in habituation studies to determine whether decreased looking times in infants are due to habituation to the stimuli or to general fatigue [38,39].

Deaf infants’ general cognitive and language skills were assessed using the Bayley Scales of Infant and Toddler Development [36] and the Preschool Language Scales– 4th Edition (PLS-4; [40]). These tests were administered by trained professionals at regular intervals following cochlear implantation as part of their long-term clinical care.

#### Habituation phase

During the habituation phase, infants observed either Object A or B over multiple trials. Each habituation trial began with the attention-grabber to orient the infants to the center of the screen, followed by the object. Trials lasted for a minimum of one second and a maximum of 20 seconds. A baseline looking time for each infant was calculated as the mean looking time during the first three habituation trials. The habituation criterion was defined as three consecutive trials in which the mean looking time to the stimulus was less than or equal to 50% of the baseline. Mean looking time for sets of three trials were calculated using a moving window of analysis (e.g., trials 1–3, 2–4, 3–5) rather than a fixed window of analysis (e.g., trials 1–3, 4–6, 7–9) to maximize the opportunity for infants to reach the habituation criteria. Stimuli presentation continued until the infant reached the habituation criterion or up to a maximum of 18 trials [34].

#### Dishabituation phase

The dishabituation phase began immediately after the habituation criterion was reached. The experimenter was blind to when the experiment transitioned from habituation to the dishabituation phase. Like the habituation trials, each trial in this phase lasted for a minimum of one second and a maximum of 20 seconds. The dishabituation phase consisted of 14 trials: four novel trials and 10 familiar trials, which were presented in four continuous blocks. Following the procedure of [34], Block 1 consisted of one novel and one familiar trial, and Blocks 2–4 each consisted of one novel trial and three familiar trials. The familiar trials were identical to the habituation trials. During the novel trials, infants observed the object that they had not seen during the habituation phase. To control for spontaneous post-habituation recovery effects, half of the infants observed the novel object first and the other half observed the familiar object first.

## Data analysis

### Habituation measures

Throughout all phases of the experiment, looking time on each trial was calculated as the total time in seconds that infants were fixated on the visual stimulus. During the habituation phase, look-away rate was calculated by offline coding of each videotaped session by two independent coders who were blind to the infant’s hearing status. A look-away was defined as a gaze shift away from the visual stimuli during a trial that did not end that trial. Because trials only ended when infants looked away for more than one second (or until the 20s maximum) infants could make brief gaze shifts away from the screen (<1s) which did not end the trial. Look-away rate per minute was calculated as the total number of looks away from the stimulus during habituation, divided by the total looking time, and multiplied by 60s [32]. Lastly, we calculated the difference in mean looking time between familiar and novel trials during the dishabituation phase to yield a score that reflects infants’ preference for the novel items.

Habituation measures included the number of trials to reach criterion, growth slope over the first four trials, look-away rate, and dishabituation score. In the literature, the number of trials to reach habituation and dishabituation—i.e., novelty preference—has been shown to predict IQ later in development (for a meta-analysis, see [41]). Look-away rate has been shown to increase across age and to be significantly negatively correlated with habituation length, suggesting that these two indices of attention are tightly coupled during infancy [32,33]. Next, to examine potential differences in the habituation slopes (i.e., the change in looking time from one trial to the next), we conducted a growth curve analysis [42] of the first four habituation trials. We selected only the first four trials because four was the fewest number of trials in which an infant could reach the habituation criterion. Therefore, all infants experienced at least four trials, while not all infants experienced five or more trials.

### Outcome measures

Receptive and expressive language skills were assessed using the Auditory Comprehension and Expressive Communication subscales of the PLS-4, which were combined to yield a composite Total Language score. The PLS-4 measures sound awareness and vocalizations for infants younger than 12 months, and additionally includes items that measure word production and object knowledge for infants between 13 and 23 months of age. The Cognitive Scale of the Bayley Scales of Infant and Toddler Development [36] was used to as a measure of general cognitive skills. Because not all infants were assessed at identical post-implantation intervals, scores were aggregated from each assessment over the first year post-CI. For example, scores from assessments conducted at 10- and 12-months post-implantation would be averaged together to yield one post-CI score.

To examine relations between outcome measures and infant habituation behavior, we conducted Pearson bivariate correlations between the raw scores at each assessment year (pre- and post-CI) and habituation measures. Based on prior research, we hypothesized that longer time to habituate and lower look-away rates would reflect slower encoding of visual stimuli and would correspond to more protracted language and cognitive growth. We therefore predicted that time to habituate and look-away rate during habituation would correlate with scores on the Bayley Cognitive subscale at the pre-CI assessment, and that they would correlate with PLS-4 total language scores at pre- and/or post-CI assessments. We adopted a Bonferroni-adjusted alpha level of 0.01 (.05/4) in the evaluation of statistical significance. For thoroughness, we report the results of correlations between all habituation measures and outcome measures at both pre- and post-CI time points.

## Results

We conducted a series of linear regression analyses to examine the effects of maternal education, unaided pure tone averages (PTA), and communication mode at time of test on habituation measures for the deaf infants. There were no effects of maternal education, communication mode, or unaided PTAs on any variable, *ps* > .34. Because our sample featured a large age range that extends beyond the typical range for habituation studies, we first examined whether results for the habituation measures differed for younger vs. older infants. To do so, we created a binary variable ‘Age’, to differentiate infants younger than 12 months vs. infants older than 12 months. Separate univariate analyses of variance (ANOVAs) were conducted for each habituation measure, with Hearing status (deaf vs. hearing) and Age (younger than 12 months vs. older than 12 months) as between-group factors. These analyses revealed no significant main effects or interactions with Age (*ps* > .18) for the number of habituation trials, mean habituation trial length, growth slopes, or dishabituation scores (*ps* > .15). Therefore, we collapsed across Age in subsequent analyses.

### Baseline visual attention

There were no differences in overall looking time between pre- and post- test looking times within each infant group separately (*ps* > .25), although some infants did show decreased looking during the post-test compared to the pre-test (n = 8 in the deaf group and n = 8 in the hearing group). There were also no differences between groups on the post-test, *p* = .46. However, deaf infants showed significantly longer looking times during the pre-test than hearing infants, *F(*1, 45) = 4.91, *p* = .03, *d* = .66. See Table 2 for descriptive statistics.

**Table 2:** Descriptive statistics.

	# Habituation Trials			Look-away Rate			Mean Looking Time											
							All Habituation Trials			Novel Test Trials			Familiar Test Trials			All Test Trials		
	M	SD	range	M	SD	range	M	SD	range	M	SD	range	M	SD	range	M	SD	range
Deaf infants	8.52	3.13	5–17	6.19	4.22	.7–13.68	69.63	47.80	31.7–200.3	7.79	3.60	2.9–16.0	5.92	3.23	1.7–13.6	90.37	44.51	36.8–193.6
Hearing infants	6.78	2.37	4–15	9.93	6.70	0–26.32	41.68	26.02	16–104.4	5.57	4.25	0.9–15.6	4.15	2.17	1.2–8.1	63.78	36.98	15.3–140.4
*F* statistic	4.40			4.22			6.06			1.91			2.18			2.20		
*p* value	.04			.05			.02			.06			.03			.03		

### Habituation phase

**Fig 2:**
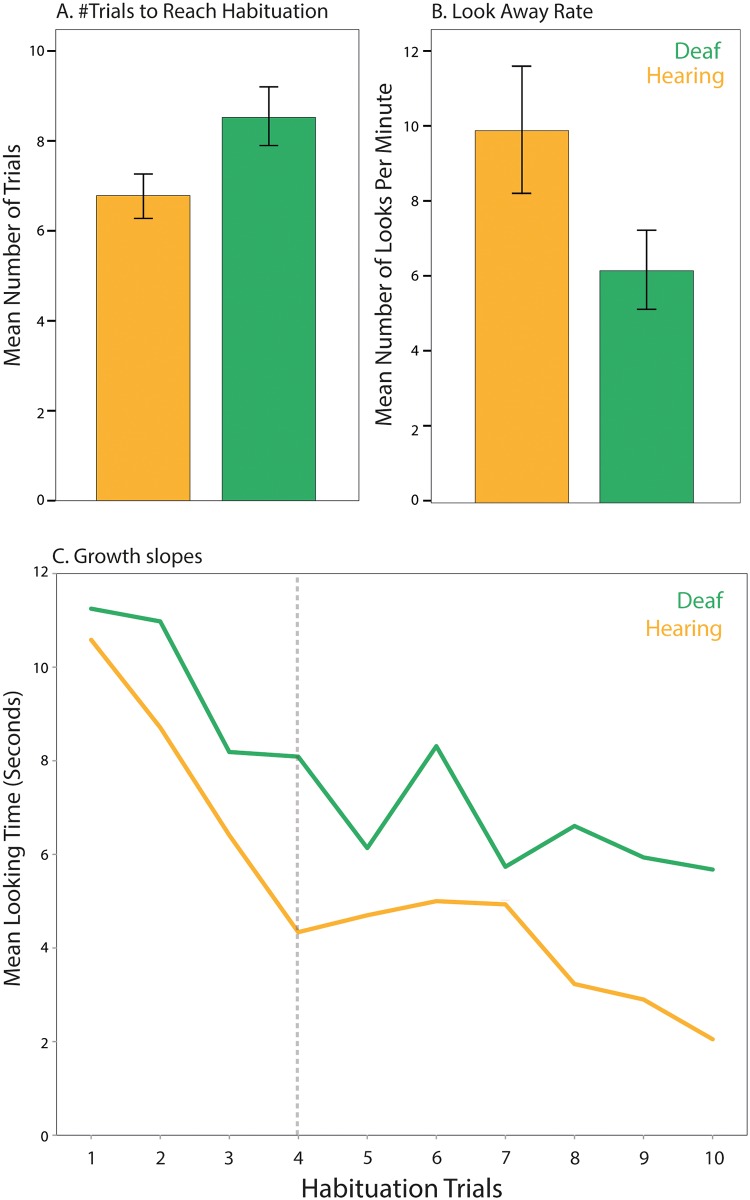
Visual habituation in deaf and hearing infants. [A] Deaf infants required more trials to reach the habituation criterion and [B] demonstrated lower look-away rates across habituation trials than their age-matched hearing peers. [C] Deaf infants demonstrated a shallower slope on the first four habituation trials. Note that data points after the fourth habituation trial are based on increasingly fewer infants and a different number of infants per group and should thus be interpreted with caution.

Deaf infants required a greater number of trials to reach the habituation criterion, *F(*1, 45) = 4.40, *p* = .04, *d* = .63 (Fig 2A), and demonstrated a lower look-away rate than hearing infants, *F(*1, 32) = 4.22, *p* < .05, *d* = .65 (Fig 2B). Total looking time and look-away rate during habituation were significantly negatively correlated across groups, *r* = -.49, *p* < .01, indicating that infants with longer looking times during habituation demonstrated lower look-away rates, consistent with previous studies [30]. The growth curve analysis revealed that the looking time slopes across the first four habituation trials significantly differed between groups, β_11_ = -.86, *p* = .02 (Fig 2C). Together, these findings suggest the deaf infants habituated more slowly to the novel object relative to the hearing infants. There were no differences in the proportion of infants (deaf vs. hearing) who did not habituate to the stimuli, *X*^*2*^ (1, n = 52) = 0.59, *p* = .44.

### Dishabituation phase

During the dishabituation phase, both groups of infants demonstrated a significant novelty preference, looking longer to the novel stimulus relative to the familiar stimulus across trials: deaf *t*(22) = 3.99, *p* < .01, *d* = 1.18, hearing *t*(22) = 2.49, *p* = .02; *d* = .73. Thus, although the deaf infants took longer to habituate to the stimuli, there were no group differences in subsequent responsiveness to a novel visual stimulus.

Differences in looking times extended throughout the dishabituation phase. Deaf infants continued to look significantly longer than the hearing infants did across the novel and familiar trials, *F(*1, 45) = 4.75, *p* = .04, *d* = .65. Together, these findings reveal that deaf infants are slower to habituate to a visual stimulus and show lower look-away rates during habituation than hearing infants, suggesting that hearing loss may be associated with differences in basic cognitive processing of visual stimuli during infancy.

### Outcome measures

**Fig 3:**
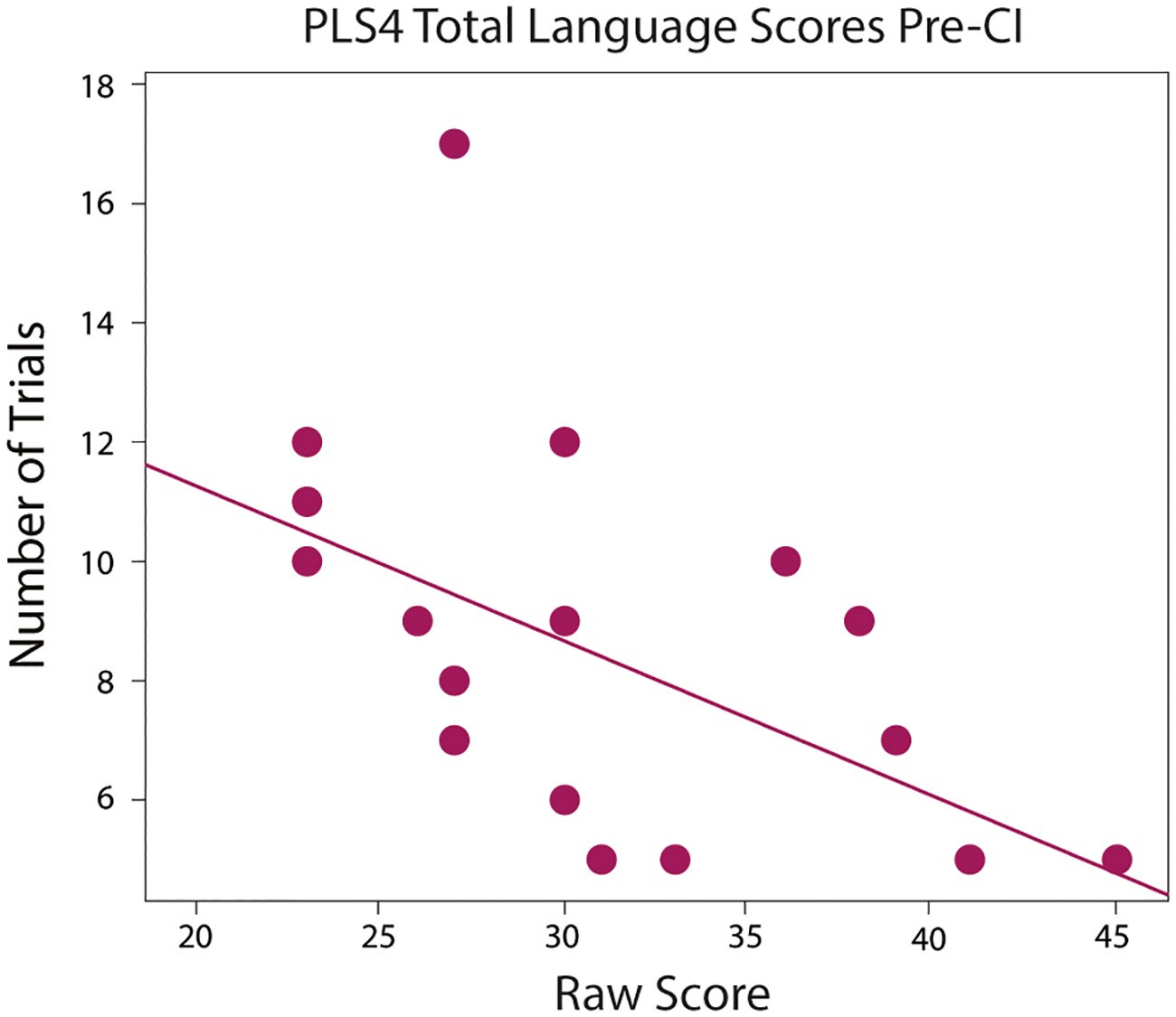
Relations between the number of trials to habituate and language scores prior to cochlear implantation.

Bivariate correlations between habituation measures and scores on the cognitive and language outcome measures for the deaf group are summarized in Table 3. The number of trials to habituate was significantly correlated with raw scores on the PLS-4 Total Language scale at the pre-implantation time point, *r* = -.45, *p* = .011, *n* = 20. Deaf infants who habituated faster to a visual stimulus displayed higher language scores (Fig 3). Look-away rate, growth slopes, and dishabituation scores did not correlate with any outcome measures pre- or post-implantation, *ps* > .29. There were no other significant correlations between habituation looking time or look-away rate and outcome measures, *ps* > .12 (Table 3).

**Table 3:** Pearson correlations between deaf infants’ visual habituation, cognitive, and language scores.

Outcome measure	Habituation Measure				
	*N*	# Trials to Reach Habituation	Growth Slopes	Look Away Rate	Dishabituation Score
Bayley Pre-CI	18	-0.34	0.11	0.10	0.18
Bayley Post-CI Year 1	18	-0.32	-0.01	0.17	0.02
PLS-4 Pre-CI Total Language Score	20	-0.56**	-0.46	0.25	0.25
PLS-4 Post-CI Year 1 Total Language Score	18	-0.27	-0.09	0.05	0.21

Note:

**p < 0.01 level. Outcome data were not available for all participants at every time point. This is common in clinical assessment batteries as children are subjected to extensive testing and sometimes cannot complete all assessments.

## Discussion

In the current study, we examined visual habituation and look-away rates in deaf and hearing infants to investigate whether the atypical sensory environment resulting from deafness is related to general processing of visual stimuli. Our data are the first to demonstrate differences in responses to visual stimuli between prelingually deaf infants and hearing infants. Specifically, deaf infants required more trials to habituate, showed a slower habituation rate, and demonstrated lower look-away rates during habituation to a visual stimulus than hearing infants. Deaf infants did not simply look longer overall throughout the experiment, as there were no differences in looking times during the first habituation trial or the dishabituation phase trials. These differences were also not likely to be due to additional impairments, as infants with a known condition that could affect cognitive development (e.g., microcephaly) were excluded from the current study. For the deaf infants, we also found that time to habituate correlated with language scores at the time of testing, indicating that infants who habituated more slowly had lower language levels. Together, these findings reveal differences in general processing of non-auditory visual information between deaf and hearing infants, at a younger age than has previously been tested. They also illustrate that prelingual hearing loss results in consequences that extend beyond the auditory system.

### Visual habituation in deaf infants

Our findings suggest that deaf infants’ visual processing is slower or less efficient than that of hearing infants. Habituation is thought to reflect memory and the speed at which infants encode a stimulus, which are predictive of later cognitive and language skills [27]. Look-away rates are a complementary measure of visual processing abilities, and have been demonstrated to increase across age in hearing infants as their processing efficiency increases [32]. It is important to note that all the deaf infants in our study did eventually habituate (and that there were no differences between groups in the number of infants who did not habituate) and displayed recognition of the novel stimulus during the dishabituation phase. This indicates that their visual habituation abilities are intact but are slower relative to the hearing infants.

Another possible interpretation of our results is that longer looking times and lower look-away rates reflect differences in sustained attention, rather than efficiency of information processing. In Quittner et a. (2012), deaf children with CIs showed faster improvement in visual attention skills—specifically, perceptual sensitivity and attentional control—over the course of a year relative to deaf children who used hearing aids [43]. The authors proposed that, in the absence of sound, deaf children learn to distribute their visual attention to their surroundings more than hearing children do. This may be because deaf individuals rely on vision to simultaneously focus on specific tasks and to monitor the environment (e.g., the ‘division of labor hypothesis’ [18]). In the current study, the stimuli presented to infants were complex objects with multiple features and colors. In accordance with this hypothesis, deaf infants may have displayed longer looking and slower habituate rates as a result of greater sustained attention to the objects.

The ability to sustain visual attention could be an advantage for the deaf infants rather than a deficit, as they may be acquiring richer representations of the visual stimuli than their hearing peers may. However, our data show that infants with fewer trials to habituate scored higher on standardized language assessments, suggesting that longer habituation times do not correspond to better spoken communication abilities. Given the established links between the development of attention and oral communication skills, our findings do not support the hypothesis that slower habituation reflects enhanced attention to the stimuli. Moreover, a review of the past 30 years of research on the visual abilities of deaf individuals suggests that their superior performance on visual tasks is best characterized by enhanced reactivity to visual events, rather than enhanced perceptual representations [44]. However, response speed advantages have only been reported in older children and adults; to our knowledge, there have been no reports of such evidence in infants or young children. On the contrary, studies on visual attention in infancy provide consistent evidence that younger deaf children exhibit lower performance levels on standard tasks of visual attention [9,45,46]. Our findings add to this body of work by suggesting that deaf infants exhibit less attentional control (lower look-away rates; [33]) and slower encoding [27] than their hearing counterparts.

Our findings are also consistent with prior evidence that habituation length and look-away rates during habituation are complimentary measures of processing efficiency [32]. Research has shown that typically-developing hearing infants who are slower to habituate to visual stimuli also demonstrate poorer cognitive outcomes [29–31,47]. In the current study, deaf infants who required more trials to habituate and had lower look-away rates scored lower on spoken language assessments than infants who took fewer trials to habituate and had higher look-away rates. Together, these findings suggest that slower habituation and lower look-away rates reflect less efficient visual processing.

### Possible mechanisms

One explanation for slower visual processing in the deaf infants is their limited auditory experiences. Limited exposure to auditory input from birth has been proposed to result in delays in general cognitive development [19]. According to the auditory scaffolding hypothesis, a possible underlying reason is that the acoustic signal provides exposure to temporal sequences of events, which is vital for the development of general processing abilities. Consistent with this hypothesis, our findings suggest that deaf infants’ processing of visual stimuli is altered relative to their hearing peers, possibly due to their limited acoustic experiences.

An alternative explanation is that deaf infants’ cognitive development may be protracted because of delayed language development. Language and cognitive skills develop interdependently and likely share common underlying mechanisms in both deaf and hearing children [48,49]. Consistent with this view, our findings show that visual habituation and language scores are correlated at the time of testing, suggesting that these abilities are already coupled in our sample of infants. Recent evidence has shown that early parent-child interactions between deaf infants and their hearing parents are influenced by their mismatch in communication modes, typically with negative consequences [50,51]. The early language environment of the deaf infants in our study was likely to be impoverished relative to that of their hearing peers, possibly reducing the opportunities for cognitive abilities to develop. Interestingly, we did not find any effects of communication mode on habituation measures, suggesting that those infants with ASL experience (who presumably have more language exposure than infants from oral-only families prior to cochlear implantation) did not demonstrate shorter habituation times than infants with no sign experience. However, given that there were only seven infants with sign experience in our sample, this should be a target for future research.

It remains difficult to untangle whether differences in visual processing in deaf infants and children result from delays in cognitive function, language exposure, or both. In prior research, one potential confound is that some of the tasks used to examine cognitive performance in deaf and hearing individuals may rely on verbal rehearsal strategies, therefore creating a natural disadvantage for the deaf group [52]. The current study is therefore unique, in that visual habituation requires no verbal rehearsal strategies. Therefore, differences in performance between deaf and hearing groups cannot be attributed to the task requirements.

To isolate the contribution of auditory experience to visual attention, independent of language, Dye & Hauser [21] conducted a study using the ‘continuous performance test’ with DOD children and hearing children. Their findings revealed that younger deaf children (6–8 years old) performed poorly on this selective attention task, but there were no differences between older (9–13 years) deaf and hearing children. All deaf children exhibited weaker cognitive control: their performance was more likely to be classified as abnormal based on published age norms. This finding suggests that the deaf children—who had all experienced a natural language from birth—still show differences in cognitive control relative to hearing children. These cognitive differences, when combined with additional language delays experienced by deaf infants born to hearing parents, may explain the wide range of nonverbal skills in which deaf children underperform compared to their hearing peers. Longitudinal research that addresses the early effects of communicative interactions and the development of early cognitive abilities has the potential to shed light on the complex interactions between language and cognition in this population.

One additional possibility to consider is that structural-functional changes in the brain could underlie differences in processing speed or efficiency. As described in the introduction, deprivation in one sensory modality results in cross-modal plasticity throughout the brain. In the case of hearing loss, there is evidence to suggest that these cross-modal changes—in which the auditory cortex assumes some of the functions of visual cortices—can result in both adaptive gains but also maladaptive outcomes [4]. For instance, speech performance outcomes in deaf CI recipients are poorer when they exhibit a greater degree of cross-modal cortical reorganization [53,54]. In deaf infants, patterns of increased connectivity between auditory and visual cortical regions could result in a more distributed functional network, which could initially delay processing speed. This interpretation is speculative, though it is consistent with current evidence and represents an interesting avenue for future research.

One limitation of the current study is the relatively wide age range of our sample, which is a common characteristic of conducting research with deaf infants prior to cochlear implantation. Because of this wide age range, the mechanisms driving visual habituation behaviors could potentially shift. However, our aim was to examine the difference in behavioral responses to unimodal visual stimuli between deaf and hearing infants, rather than to make claims about the potential physiological mechanisms underlying visual habituation. Future work should replicate these findings with a narrower age range.

## Conclusion

This study reveals differences in visual habituation, a building block of cognitive development, between deaf and hearing infants. Deaf infants required a greater number of trials to habituate, showed slower rates of habituation, and had lower look-away rates during habituation than age-matched hearing infants. These data provide converging evidence for slower or less efficient processing of the visual stimuli than their hearing counterparts. In addition, these measures of visual habituation correlated with the deaf infants’ performance on standardized tests of language skills. Together, our findings provide the first evidence to suggest that differences in processing visual stimuli between deaf and hearing children emerge early in infancy, prior to advanced language acquisition. Our findings also support the notion that early differences in visual information processing may explain some of the variability in deaf children’s neurocognitive development. Given the predictive relationship between habituation behaviors in infancy and later cognitive development [29,47], it is especially important for future research to investigate the effects of hearing loss on cognitive development.

## Supporting information

S1 File Final dataset. (SAV)
